# Neuroendocrine Differentiation of Lung Cancer Cells Impairs the Activation of Antitumor Cytotoxic Responses in Mice

**DOI:** 10.3390/ijms24020990

**Published:** 2023-01-04

**Authors:** Ricardo Fosado, Jazmín E. Soto-Hernández, Rosa Elvira Núñez-Anita, Carmen Aceves, Laura C. Berumen, Irasema Mendieta

**Affiliations:** 1 Posgrado en Ciencias Químico-Biológicas, Facultad de Química, Universidad Autónoma de Querétaro, Cerro de las Campanas S/N, Querétaro 76010, Mexico; 2 Facultad de Medicina Veterinaria y Zootecnia, Universidad Michoacana de San Nicolás de Hidalgo, Tarímbaro 58893, Mexico; 3 Instituto de Neurobiología, Universidad Nacional Autónoma de México-Campus Juriquilla, Boulevard Juriquilla 3001, Juriquilla, Querétaro 76230, Mexico

**Keywords:** lung cancer, adenocarcinoma, neuroendocrine cancer, neuroendocrine differentiation, immunosuppression, cytotoxic cells, immune escape

## Abstract

Lung cancer has the highest mortality among all types of cancer; during its development, cells can acquire neural and endocrine properties that affect tumor progression by releasing several factors, some acting as immunomodulators. Neuroendocrine phenotype correlates with invasiveness, metastasis, and low survival rates. This work evaluated the effect of neuroendocrine differentiation of adenocarcinoma on the mouse immune system. A549 cells were treated with FSK (forskolin) and IBMX (3-Isobutyl-1-methylxanthine) for 96 h to induce neuroendocrine differentiation (NED). Systemic effects were assessed by determining changes in circulating cytokines and immune cells of BALB/c mice immunized with PBS, undifferentiated A549 cells, or neuroendocrine A549_NED_ cells. A549 cells increased circulating monocytes, while CD4^+^CD8^−^ and CD4^+^CD8^+^ T cells increased in mice immunized with neuroendocrine cells. IL-2 and IL-10 increased in mice that received untreated A549 cells, suggesting that the immune system mounts a regulated response against adenocarcinoma, which did not occur with A549_NED_ cells. Cocultures demonstrated the cytotoxic capacity of PBMCs when confronted with A549 cells, while in the presence of neuroendocrine cells they not only were unable to show cytolytic activity, but also lost viability. Neuroendocrine differentiation seems to mount less of an immune response when injected in mice, which may contribute to the poor prognosis of cancer patients affected by this pathology.

## 1. Introduction

Lung cancer has the highest mortality rate among all types of cancer worldwide and is also the second-most diagnosed malignant disease in both men and women [1]. Despite the implementation of new treatments, the 5-year survival rate for this type of cancer is still less than 15% in developed countries, whereas this figure drops to 5% in developing countries [2,3].

Lung cancers with epithelial origin can be broadly classified into two types: small cell carcinomas (SCLC) and non-small cell carcinomas (NSCLC) [4]. Amongst all subtypes of NSCLC, adenocarcinoma is the most common (~40% of all cases), arising from epithelial cells that produce fluid or mucus [2,3,5].

During tumor progression, and as a result of evolutionary pressures within the tumor microenvironment, some cells may undergo a process of neuroendocrine differentiation (NED), acquiring neural and endocrine properties. Up to 50% of NSCLC cases may contain a large number of focal subpopulations of cells that have undergone NED, being more abundant in higher-grade tumors [6], and although these cells prevail among a predominant population of non-differentiated adenocarcinoma cells, they have a great effect on the tumor microenvironment and cancer progression [7,8].

The complexity of NED in cancers has made it difficult to associate it with a specific prognosis for patients; however, the presence of its markers in prostate carcinomas has been widely correlated with an increase in metastasis, pathological progress, and lower survival of patients [9,10,11].

Cancer cells that acquire this phenotype can produce neurotransmitters and growth factors that favor tumor growth through paracrine stimulation. For example, IL-8, GRP, neurotensin, and serotonin are molecules produced by neuroendocrine cells whose oncogenic and mitogenic activity is well-documented [7,9,12]. Neuroendocrine cells also express high levels of anti-apoptotic molecules, such as survivin and Bcl-2 family proteins, so it seems that NED provides resistance to programmed cell death [10].

Another essential characteristic of neuroendocrine cancer cells is that they are in a post-mitotic (non-proliferative) state, and their differentiation can be complete, partial, and even reversible [7,10]. The neuroendocrine cells’ low proliferative capacity significantly impacts tumor progression, especially in response to chemotherapy, which commonly targets cells that multiply rapidly and uncontrollably, endowing these cells with chemoresistance [12]. Therefore, the reversibility of NED represents yet another challenge for patients since neuroendocrine cells surviving anticancer drugs can resume proliferation and cause tumor recurrence. 

Recently, using a lung adenocarcinoma cell line (A549), it was shown in vitro that the cytolytic capacity of Jurkat cells (T lymphocytes) decreases substantially when confronted with cancer cells differentiated to a neuroendocrine phenotype [13]. This led us to suggest that, in addition to all the protumorigenic properties of neuroendocrine cells, the factors that they secrete may serve as immunomodulators, interfering with the immune system’s response against cancer and adding to the plethora of reasons explaining why this is a more aggressive form of cancer.

This work aimed to determine the effect of neuroendocrine differentiation of lung cancer cells on the immune system of mice, particularly on peripheral blood mononuclear cells (PBMCs), which comprise most of the immune cells that respond against cancer, i.e., monocytes, dendritic cells, NK cells, T cells, and B cells.

Our results confirm that mice immune cells are unable to kill lung cancer cells with neuroendocrine differentiation, contrary to what happens when they are confronted with their undifferentiated counterparts—inducing cancer cell death. We also proved that, somehow, neuroendocrine cells inhibit the cell proliferation of immune cells, suggesting a proapoptotic or an exhaustion activity of the immune cells. Finally, based on PBMC and cytokine profiles, we hypothesize that neuroendocrine differentiation promotes the escape from the immune system, mainly through immunosuppression mediated by T cell polarization.

## 2. Results

### 2.1. Neuroendocrine Differentiation of A549 Lung Cancer Cells Is Induced by Treatment with cAMP-Elevating Agents

**Figure 1:**
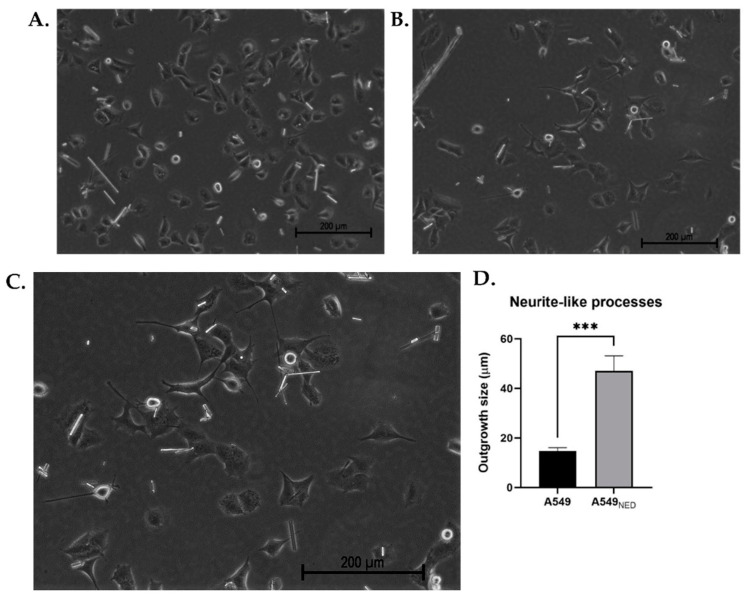
Morphological changes in A549 cells treated with FSK and IBMX. Undifferentiated A549 cells show normal epithelial morphology (**A**) whereas A549_NED_ cells present neurite-like outgrowths 24 h after treatment with cAMP-elevating agents (**B**). A zoomed-in image of the cells with the largest processes observed in (**B**) is also included (**C**). The bar graph shows the average outgrowth size in treated and untreated cells (**D**); results are presented as the mean ± SEM (n = 3, *** *p* < 0.001).

To induce a neuroendocrine phenotype, A549 cells were treated with FSK (0.5 mM) and IBMX (0.5 mM). Treated cells presented neurite-like processes, unlike control A549 cells (Figure 1). After measuring the size of the neurite-like outgrowths in neuroendocrine A549_NED_ cells and comparing them with epithelial outgrowths from untreated cells, it was found that there was a significant difference (*p* = 0.0004) between both groups, with neurite-like outgrowths being significantly longer in neuroendocrine cells (Figure 1D). Outgrowths present in A549 cells measured 14.7 ± 1.36 µm, while treated cells had processes that reached an average size of 47.02 ± 6.15 µm.

**Figure 2:**
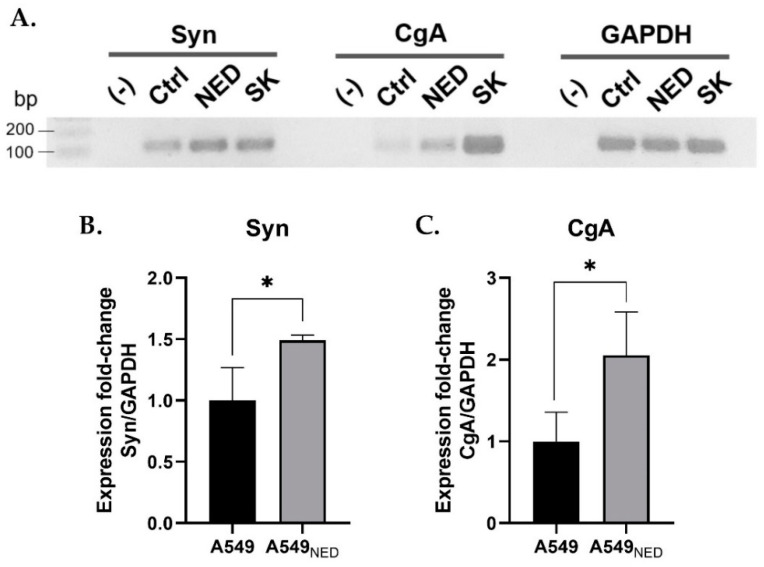
Gene expression of neuroendocrine markers. Syn, CgA, and GAPDH were amplified by RT-PCR from cDNA of A549 cells without treatment (Ctrl), A549 treated cells (NED), and neuroblastoma SK-N-AS cells (SK) as a positive control (**A**). Bar graphs showing changes in the relative gene expression of Syn (**B**) and CgA (**C**) in A549 cells with or without neuroendocrine differentiation (n = 3; * *p* < 0.05).

To further confirm that cells were successfully differentiated, we measured chromogranin A (CgA) and synaptophysin (Syn), which are common neuroendocrine markers. Semi-quantitative analysis was performed using RT-PCR and densitometry, determining the relative expression of the genes of interest. As depicted in Figure 2A, reaction products corresponded to the expected sizes for each gene (Syn, 123 bp; CgA, 102 bp; GAPDH, 120 bp). Data were normalized using GAPDH and the control group, obtaining the fold change in relative expression. Both Syn and CgA had a significantly higher expression in A549_NED_ cells (*p* = 0.0356 and *p* = 0.0467, respectively); on average, A549_NED_ cells had a 1.491-fold increase in Syn expression, whereas CgA expression increased 2.051-fold (Figure 2). These results confirm that treatment with FSK and IBMX induced a neuroendocrine phenotype in A549 cells.

### 2.2. CD68^+^ Monocyte Levels Increase and CD4^+^ T Cells Decrease in Mice Immunized with A549 Cells, whereas Double Positive CD4^+^CD8^+^ T Cells Increase in Mice Immunized with A549_NED_ Cells

Cell populations within PBMCs were analyzed with flow cytometry. An antibody against CD68 was used to identify monocytes, a population of cells that establishes in the tissues as macrophages after circulating through the vasculature. Anti-CD4 and anti-CD8a antibodies were used to differentiate populations between helper and cytotoxic T cells, respectively. Finally, CD335 was used to identify NK cells—cytotoxic members of the innate immune system that play a pivotal role in protecting the body against cancer. 

**Figure 3:**
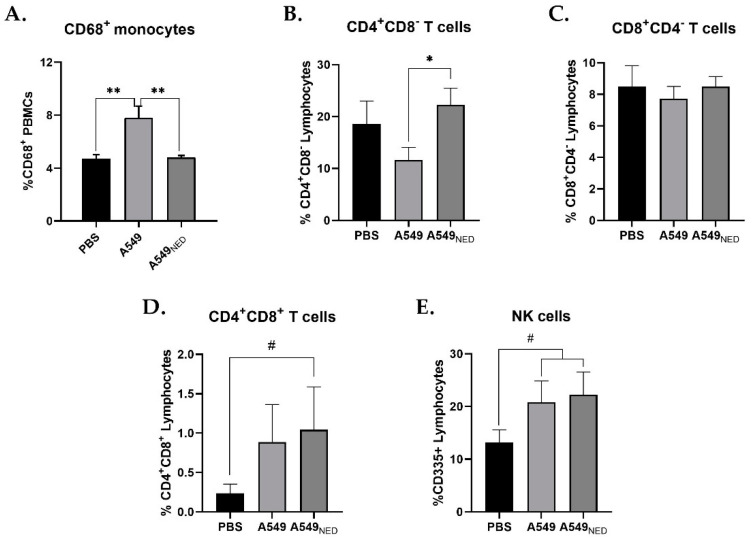
Changes in PBMC profiles after immunization. Flow cytometric analysis of PBMC samples obtained from the different groups of mice, targeting CD68 (a monocyte/macrophage cellular marker), CD4 (a co-receptor for the T cell receptor and specific marker of T helper cells), CD8a (a co-receptor for the T cell receptor and specific marker of cytotoxic T cells), and CD335 (a cytotoxicity-activating receptor that mediates tumor cell lysis and distinguishes NK cells from other populations). The bar graphs show the percentage of CD68^+^ (**A**), CD4^+^ (**B**), CD8^+^ (**C**), double positive CD4^+^/CD8^+^ (**D**), and CD335^+^ (**E**) cells in PBMCs from the different groups of mice. Results are presented as the mean ± SEM (n = 4, ** *p* < 0.01, * *p* < 0.05, # *p* < 0.1).

The analysis showed that, in mice immunized with undifferentiated A549 cells, the percentage of circulating monocytes increased compared to the control group, while monocytes in mice immunized with A549_NED_ cells remained at baseline levels (Figure 3A). Statistical analysis showed that there was a significant difference between the groups (*p* = 0.0045), while the mice immunized with PBS and A549_NED_ were statistically identical.

In mice that were immunized with undifferentiated A549 cells, the percentage of CD4^+^ T lymphocytes decreased compared to the group immunized with A549_NED_ cells (Figure 3B). Statistical analysis showed a significant difference between the immunized groups (*p* = 0.0217). Again, the data indicated that there was no statistically significant difference between the groups immunized with PBS and A549_NED_ cells.

Unexpectedly, cytometric analyses targeting CD8a showed that, in both groups of immunized mice, the percentage of CD8^+^ T lymphocytes remained the same as in the control group (Figure 3C). Statistical analysis did not show differences between groups. 

In mice that were immunized with differentiated A549_NED_ cells, the percentage of CD4^+^/CD8^+^ double-positive T lymphocytes increased compared to the control group injected with PBS (Figure 3D). Statistical analysis showed a quasi-significant difference between the A549_NED_ and PBS groups (*p* = 0.0988), whereas the data indicated that there was no significant difference between the PBS and A549 groups.

Finally, mice that were immunized with either undifferentiated or neuroendocrine A549 cells had more circulating NK cells compared to the control group (Figure 3E). The data indicated that there was no statistically significant difference between the groups immunized with A549 and A549_NED_ cells.

**Figure 4:**
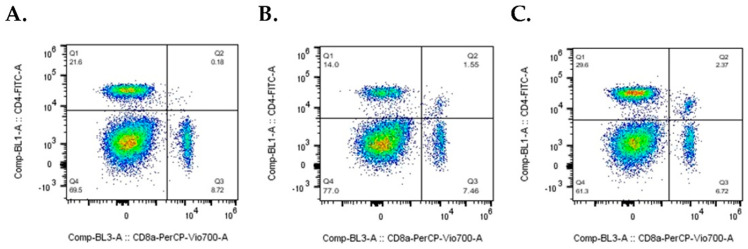
Changes in T cell populations. Flow cytometer results from individual samples are presented; dot plots were generated from a mouse injected with PBS (**A**), A549 cells (**B**), and A549_NED_ cells (**C**). Q1 contains CD4-positive cells, Q2 contains CD4/CD8 double-positive cells, Q3 encompasses CD8 positive cells, and Q4 includes cells that are negative for both CD4 and CD8.

Figure 4 shows representative individual PBMC samples from immunized mice, showing a decrease in CD4^+^ T cells in those immunized with A549 cells (Figure 4B, Q1), and an increase in CD4^+^CD8^+^ double-positive T cells in mice injected with neuroendocrine cells (Figure 4C, Q2).

### 2.3. IL-2 Increases in Mice Immunized with A549 Cells, whereas IL-10 Decreases in Mice Immunized with A549_NED_ Cells and IFN-γ Stays the Same in All Groups

To identify a systemic inflammatory profile in the immunized mice, cytokine levels in serum and plasma were determined with ELISA, measuring IL-2 and IFN-γ, associated with pro-inflammatory activity, and IL-10, commonly associated with anti-inflammatory activity.

**Figure 5:**
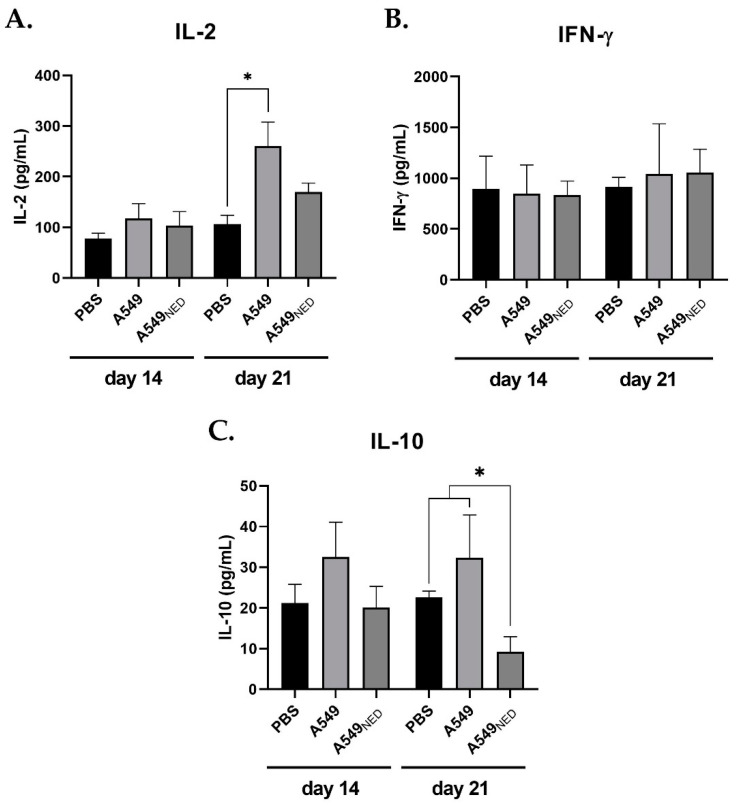
Cytokine levels in immunized mice. The bar graphs show IL-2 (**A**), IFN-γ (**B**), and IL-10 (**C**) levels 14 and 21 days after the first immunization; data are shown in pg/mL (n = 4, * *p* < 0.05).

Fourteen days after the first immunization, there were no statistical differences in IL-2, IL-10, and IFN-γ levels between the groups (Figure 5). However, 21 days after the first immunization, there was a significant difference (*p* = 0.0165) between the concentration of IL-2 in the group of mice immunized with PBS (106.66 pg/mL) and the group immunized with A549 cells (260.07 pg/mL) (Figure 5A). Surprisingly, IFN-γ levels showed no differences between groups even after 21 days (Figure 5B). Interestingly, 21 days post-immunization, IL-10 levels decreased significantly in mice immunized with A549_NED_ cells, averaging 9.223 pg/mL, as opposed to the control group and mice immunized with A549 cells, with means of 22.61 and 32.35 pg/mL, respectively (Figure 5C).

### 2.4. PBMCs Exert Cytotoxic Activity on A549 Cells, but They Lose This Capacity when Confronted with A549_NED_ Cells

To determine whether PBMCs could kill A549 or A549_NED_ cells ex vivo, we cocultured them and then we measured the cell viability of both cancer and mononuclear cells. 

**Figure 6:**
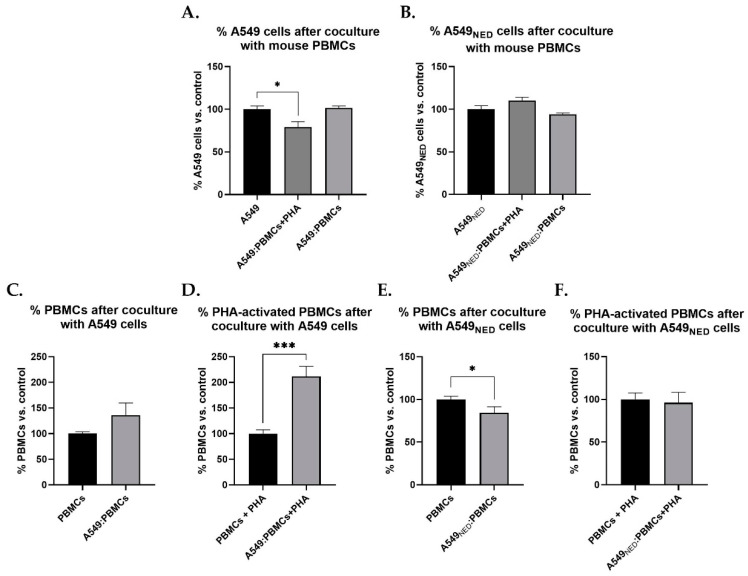
Adenocarcinoma and mononuclear cells after cocultures. The number of A549 (**A**) and A549_NED_ (**B**) cells is expressed as a percentage relative to the negative control—cells without coculture. Cells were cocultured at a 1:2 ratio (A549:PBMC) with or without PBMC pre-treatment using phytohemagglutinin (PHA). Untreated (**C**) or PHA-activated (**D**) PBMC number after coculture with A549 cells is also shown, as well as the number of untreated (**E**) or PHA-activated (**F**) PBMCs after coculture with A549_NED_ cells, all expressed as a percentage relative to the negative control. Cells were cocultured at a 1:2 ratio (A549:PBMC) (n = 3, * *p* < 0.05, *** *p* < 0.001).

When undifferentiated A549 cells were cocultured with phytohemagglutinin (PHA) pre-activated PBMCs, A549 cells decreased by 21.2% compared to the negative control without coculture (Figure 6A). Data analysis showed that this difference was statistically significant (*p* = 0.028). Coculture of A549 cells with PBMCs without pre-activation was also evaluated; however, in this case, the number of cancer cells stayed the same as the control group (Figure 6A). This means that only PHA-activated PBMCs could kill A549 cells.

In contrast to what was observed for undifferentiated A549 cells, when coculturing A549_NED_ cells with PHA pre-activated PBMCs, neuroendocrine cancer cell number did not decrease; interestingly, it increased by 10.16% compared to control cells (Figure 6B). 

These results imply that PBMCs cannot kill adenocarcinoma cells with a neuroendocrine phenotype, contrary to those in their undifferentiated state. Also, interactions between the different cell types in the coculture somehow promoted the proliferation of cells that remained without terminal differentiation.

### 2.5. PBMCs Proliferate in Response to A549 Cells but Die when Cocultured with A549_NED_ Cells

After coculturing PBMCs with A549 cells, a 36.34% increase in the number of PBMCs could be observed (Figure 6C). Despite the percentage change between these two groups, statistical analysis indicated that the differences were not significant. However, when confronting PHA-activated PBMCs with A549 cells, mononuclear cell numbers increased by 111.96%; that is, the population of PBMCs doubled in the presence of A549 cells (Figure 6D). Data analysis showed that the differences were statistically significant (*p* = 0.0006).

In contrast to what was observed in the previous group, when PBMCs were cocultured with A549_NED_ cells, mononuclear cells decreased by 15.51%, i.e., neuroendocrine cells induced PBMC death (Figure 6E). Statistical analysis indicated that the difference was significant (*p* = 0.0329). When PHA-activated PBMCs were cocultured with A549_NED_ cells, only a slight decrease in cell number could be observed (Figure 6F). However, in this case, the statistical analysis indicated that the differences observed were not statistically significant.

## 3. Discussion

The impact of neuroendocrine marker expression on the survival of patients with lung cancer is still controversial. The results from different studies are conflicting: some have found that NE differentiation has a negative impact on survival, but others have failed to demonstrate any correlation with prognosis [14]. Nevertheless, these data are difficult to interpret because the proportion of NE-differentiated NSCLCs varies according to the technique and marker used, and because the respective patient subsets were small. A correlational study with a larger cohort and more sensitive markers (such as INSM1), are needed to establish a substantial prognostic impact [15].

Cerasuolo stated that the ability of neuroendocrine cells to induce an ‘early onset of a hormone-refractory status’ is intriguing and clinically relevant. Therefore, the data on the differential pattern of immunomodulator production support the idea that immunomodulators can locally act by promoting paracrine interaction with the tumor microenvironment, generating worse prognostic outcomes for patients [16].

Monocytes are known to have a great influence on cancer progression, participating in the antitumor response and affecting processes such as angiogenesis and metastasis [17]. Our results showed that mice immunized with A549 cells had higher levels of circulating monocytes. Even though the increase in monocyte levels has been linked to more aggressive tumors and lower patient survival with multiple types of cancer [17,18,19,20], these immune cells comprise heterogeneous populations whose response depends on the stimulus: cancer type, tumor microenvironment, and tumor stage [21]. It is believed that the increase in tumor-promoting monocytes and its correlation with aggressiveness derived from their reprogramming within the tumor microenvironment, since it has been shown that circulating monocytes—mainly non-classical ones—can produce tumoricidal factors and pro-inflammatory cytokines, present antigens, and recruit NK cells [21,22,23,24]. In our experimental model, tumors were not established, avoiding the circulating monocyte reprogramming. Therefore, we can assume that they had antitumor activity and responded against cancer cells; however, more studies are needed to confirm this, including CD47 expression in cancer cells, as a surface protein that interacts with monocytes [25,26,27].

Regarding IL-10 levels, reports indicate that CD169+ monocytes are activated cells that have an enhanced ability to stimulate cytotoxic activity in CD8^+^ T lymphocytes, and the level of these monocytes rises in the circulation of patients producing high levels of IL-10 [28]. Furthermore, activated classical and intermediate monocytes are among the most important producers of IL-10 in inflammatory contexts. We could infer a relationship between these two factors, considering that monocytes are one of the main sources of cytokines in the blood [23,29].

IL-2 has a great activating effect on monocytes, leading them to secrete growth factors and proinflammatory cytokines such as IL-6 [30]. Even monocyte exposure to IL-2 is sufficient for them to exhibit microbicidal and tumoricidal activity [31]. This information led us to suggest that the monocytes that increased in mice immunized with A549 cells had proinflammatory and anticancer activity since in this same group there was a significant increase in circulating IL-2 levels.

Furthermore, our results showed a significant difference in CD4^+^ helper T cell levels between mice immunized with A549 and A549_NED_ cells, identifying a higher percentage in the latter. These cells can organize immune responses through the secretion of cytokines by differentiating into several subtypes: the most studied Th1 and Th2, as well as Th9, Th17, Th22, and induced Tregs [32]. However, our results point to an immunosuppressive response induced by A549_NED_ cells; therefore, we hypothesize that the observed CD4^+^ T cell increase is the result of high numbers of lymphocytes polarized both to Th2 and induced Tregs.

For many years, T cell polarization to Th2 has been considered a promoter of tumor development since these cells suppress the ability of lymphocytes to polarize towards a Th1 phenotype with antitumor inflammatory activity [33,34,35]. On the other hand, Treg cells, which are also positive for CD4, have immunosuppressive activity, inhibiting cytotoxic T lymphocytes (CTLs) through molecules such as TGF-β. Studies have found an inverse correlation between the abundance of CD4^+^ Tregs and inflammatory monocytes in mice [36], which occurs with the observed monocyte increase in the group of mice immunized with A549 cells, in which CD4^+^ T cells decreased. 

On the other hand, our results suggest that CD4^+^ cells in mice immunized with A549 cells were polarized towards a Th1 phenotype, which caused an increase in the cytotoxic response correlating with favorable immunological effects [37]. It is known that Th1 lymphocytes have a great capacity to produce proinflammatory cytokines such as IL-2, promoting the cytotoxic activity of effector cells, e.g., NK cells and CTLs [33,38]. Simultaneously, IL-2 favors Th1 polarization, creating a positive feedback loop [39]. This agrees with our results since mice immunized with A549 cells showed elevated IL-2 levels, and PBMCs had improved cytotoxic capacity when confronted with undifferentiated cells.

The cytotoxic activity against tumor cells is largely mediated by CD8^+^ CTLs, as they can directly recognize cancer cells after antigen presentation and then induce apoptosis through different mechanisms [40]. Therefore, we would have expected this population to increase in mice immunized with A549 cells since this group showed a greater immune response. However, this is not what we observed, for no differences were found in the percentage of CD8-positive T cells between immunized groups.

Once the immune system eliminates a threat, effector T CD8^+^ cells contract, leaving behind a small subset of antigen-specific memory lymphocytes, which can induce a faster and more aggressive response to the antigen if it is ever recognized again [41]. Since we determined cell populations only at the end of the experiment—14 days after the second immunization—by this time the immune system may have already gotten rid of the cancer cells, contracting effector the CD8^+^ T lymphocytes responsible for the cytotoxic response. 

An alternative explanation for our results depends on IL-2, a protein that regulates key aspects of CD8^+^ T cell biology, including survival, proliferation, differentiation, cytotoxic activity, and memory cell generation [42]. However, it has been known for decades that this cytokine can also have a dual effect, promoting an activation-induced cell death program in T cells [43]. Now, recent studies have shown that continuous exposure of CD8^+^ T cells to high levels of IL-2 leads to lymphocyte exhaustion, losing reactivity to cancer cells, and therefore being unable to exert their cytotoxic function, eventually leading to their death [44,45,46]. In our study, if CTLs lost their functionality throughout the experiment due to continuous exposure to IL-2 (since its levels were high in the A549 group from day 14), by the time of cytometric analysis, these lymphocytes would have died. To corroborate this assumption, it would be necessary to determine leukocyte populations at different time points, looking for the moment when clonal expansion occurs. However, it is important to emphasize that T cell depletion mediated by IL-2 does not contradict its role in antitumor activity, since this cytokine is essential to promote CTL activity and proliferation during the initial stages of tumor development. Therefore, we cannot rule out that the cytotoxic response observed in cocultures is partially mediated by CD8^+^ lymphocytes since the time these cells were kept in the presence of A549 was shorter, avoiding overexposure to IL-2 and exhaustion of CTLs. 

NK cells play a fundamental role in the first line of defense against cancer cells, and their activity is regulated by multiple inhibitory and activating receptors [47]. In our experiments, NK cell levels increased in both groups immunized with A549 cells. 

We can assume that the cytotoxic effects observed in cocultures were mainly orchestrated by NK cells since they can directly recognize and respond to a threat without the need for antigen-presenting cells. Moreover, NK cells depend on IL-2 to expand and induce an effector response [48] and treating them with this cytokine leads to greater cytotoxic and antitumor activity [49]. Since mice immunized with A549 cells had a significant increase in IL-2 levels, we believe that NK cells in these mice were able to kill cancer cells.

Furthermore, NK cells are known to produce different cytokines, such as GM-CSF, IFN-γ, TNF-α, IL-10, IL-5, and IL-13 [50]. We did not detect higher IFN-γ levels in the A549 group, but an increase in IL-10 was observed, and although this cytokine is produced by different cell types, NK could have been an important source of the interleukin detected. IL-10 is a potent activator of NK cell proliferation and cytotoxic function in the presence of IL-18, which creates a positive feedback loop [50,51]. This could be another reason for the decreased IL-10 levels in mice challenged with A549_NED_ cells, since these cells may somehow activate regulatory mechanisms aimed at limiting the availability of molecules that promote the antitumor response.

Despite having also detected higher NK cell numbers in mice immunized with A549_NED_ cells, neither IL-2 nor IL-10 levels increased in this group. Both cytokines are important in the NKs’ cytotoxic response context, and it has been reported that their proliferation does not depend on IL-2, but their effector function does [52]. Therefore, this information suggests that NK cell activity in mice immunized with A549_NED_ was not optimal, even if they expanded. It has been reported that by limiting IL-2 availability, Treg cells negatively regulate NK cell activity [48], which is consistent with our assertion that CD4^+^ T cells polarize towards Th2 and Tregs in this group of mice.

Finally, NK cells can be forced by cancer cells to partially lose their phenotype and turn into myeloid suppressor cells (MDSC), contributing to immunosuppression [53]. This situation might explain the increase we observed in NK cells in the A549_NED_ group; however, since IL-2 is sufficient to prevent the conversion of NK to MDSC [53], this process could not have happened in mice immunized with A549 cells, where this cytokine’s levels increased considerably.

Other reports support the explanation of the results regarding cytokines. Chronic inflammation and the persistent presence of proinflammatory cytokines are correlated with a higher incidence of cancer, as well as a worse prognosis for patients [54]. In this sense, molecules that regulate systemic inflammation are needed; thus, IL-10′s anti-inflammatory activity would be necessary to negatively regulate immune responses [55,56]. For example, it is known that IL-10 inhibits the proliferation of CD4^+^ T lymphocytes and it suppresses the secretion of cytokines by Th1 cells, such as IFN-γ [55], which is consistent with our results in mice that received A549 cells: high IL-10 levels, less CD4^+^ T cells, and no IFN-γ increase. Therefore, high levels of IL-10 imply the presence of a physiological process aimed at restoring homeostasis, especially if aggressive inflammatory events occurred, suggesting that the immune response against A549 cells is regulated.

Cocultures showed that mononuclear cells were able to exert their cytotoxic effect on A549 cells. This activity may have been mediated by all the different populations of immune cells that comprise PBMCs. For example, monocytes can kill cells through cytokines and phagocytosis [21,57], and given that these cells increased in mice immunized with undifferentiated cells, we assume that they recognized and killed them. NK cells are among the most aggressive and effective cells against cancer [52], which is why we suggest that they also played a part in the observed cytotoxicity. Cytotoxic CD4^+^ and CD8^+^ T cells could also have decreased the cancer cells’ viability. In addition, we observed a decrease on the cancer cell viability observed in cocultures with PBMCs that had been pre-activated with PHA. PHA is a lectin known for decades as a mitogenic and activation agent of T lymphocytes triggering a cytotoxic response [58,59]. 

Activation of T lymphocytes before coculture seems to have been a key factor for PBMCs to demonstrate cytotoxic activity, which makes sense considering that treating T cells with PHA induces IL-2 production [60], an essential requirement for cytotoxic responses. Pre-activation may have also favored the clonal expansion of antigen-specific cells since all the components needed for this process were there from the start. We do not rule out the possibility that by coculturing cells for a longer time, natural processes would generate all the elements necessary to exert cytotoxic functions, producing cytokines that allow the effector action of monocytes and NK cells, polarizing lymphocytes to a Th1 phenotype, and generating antigen-specific cytotoxic T cells.

As expected, cocultures with A549_NED_ cells inhibited PBMCs’ cytotoxic activity, agreeing with our previous results that suggested that the immune response was impaired. Interestingly, not only were mononuclear cells incapable of killing cancer cells with a neuroendocrine phenotype; instead, the A549_NED_ might take advantage of factors secreted by leukocytes to promote their own growth, as is often seen in cancer patients. Altogether, these results support the hypothesis that T cells were polarized to Th2 and Treg phenotypes in the presence of A549_NED_ cells since these cells can produce cytokines such as IL-4 and IL-13 (Th2) and TGF-β (Treg), whose ability to promote cell proliferation has been widely documented [52,61,62,63]. 

Cocultures with A549 cells caused the proliferation of PBMCs, which was expected considering that the activation of immune cells is normally associated with their proliferation, especially in T cells [13]. Interestingly, when PBMCs were cocultured with A549_NED_ cells, mononuclear cell viability decreased significantly; this implies that neuroendocrine cancer cells could somehow induce the death of immune cells. 

**Figure 7:**
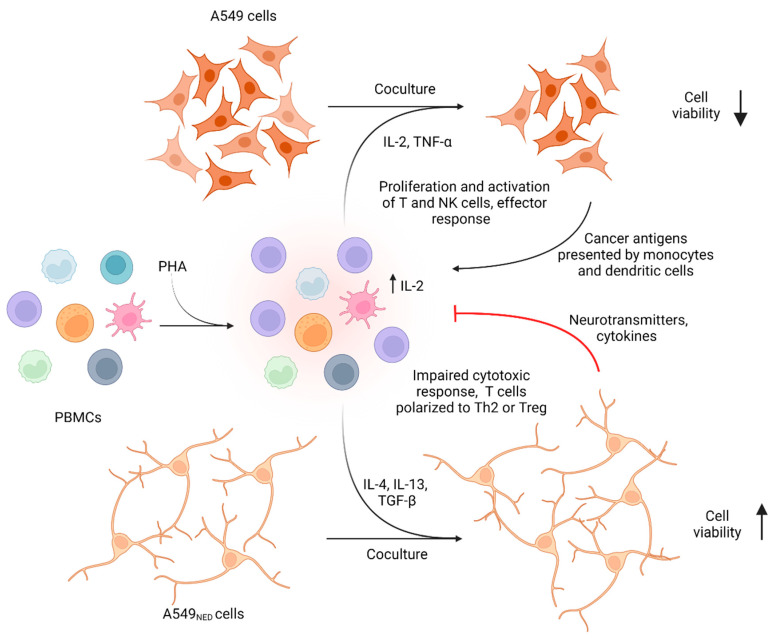
Cellular mechanisms involved in cocultures. We illustrate the mechanisms proposed to explain our results, in which A549 cells lost viability in coculture with PHA-preactivated PBMCs, while A549_NED_ cells had higher viability. Our results suggest that the pre-activation of cells with PHA caused the production of IL-2, a cytokine necessary for the activation of NK cells and monocytes, which exerted their effector functions on A549 cells, producing more proinflammatory cytokines; apoptosis favored the presentation of antigens mediated by monocytes and dendritic cells, allowing the activation and clonal expansion of cytotoxic T lymphocytes, which also exerted their cytolytic activity. A549_NED_ cells, possibly through neurotransmitters such as serotonin and norepinephrine and cytokines such as IL-4, caused the polarization of T cells towards Th2 and Treg, inhibiting cytotoxic activity and secreting cytokines that favor proliferation from cancer cells, such as IL-4, IL-13, and TGF-β [21,52,57,60,61,62]. Image created on BioRender.com, licensed and published under agreement MH24DCXFXU.

Figure 7 summarizes the proposed mechanisms involved in the response observed in cell cocultures, in which the PBMCs pre-activated with PHA were able to kill only A549 cells, but not A549_NED_ cells, which even had the ability to proliferate in the presence of mononuclear cells.

Overall, our results agree with those reported by our group in 2018, where cocultures between cytotoxic T lymphocytes (Jurkat cell line) and A549 or A549_NED_ cells also showed that cytotoxic activity decreases in the presence of neuroendocrine cells, but not in the presence of undifferentiated cancer cells. Moreover, in this study, lymphocyte viability also decreased in the presence of A549_NED_ cells, suggesting that neuroendocrine factors secreted by the cells can induce immune cell death [13].

It has been demonstrated that A549 cells with neuroendocrine phenotype produce increased levels of serotonin, while dopamine production is reduced to undetectable levels [13]. This is important given that all immune cells express serotonergic and dopaminergic components, responding in different ways to this neurotransmitter. Table 1 summarizes the effect of different neurotransmitters on immune cells, proposing why neuroendocrine cells overproduce serotonin and deplete dopamine secretion. Based on the literature, these results led us to suggest that A549_NED_ and other neuroendocrine cells overproduce other immunosuppressive factors (Table 1).

## 4. Materials and Methods

### 4.1. Neuroendocrine Differentiation of Lung Adenocarcinoma Cells

A549 cells (Cell Biolabs, San Diego, CA, USA) were cultured in DMEM (Gibco Thermo Scientific, Waltham, MA, USA) supplemented with 10% fetal bovine serum (FBS), penicillin (100 IU/mL), and streptomycin (100 μg/mL) and passaged every third/fourth day maintaining optimal confluency (~80%).

For neuroendocrine differentiation, 2.5 × 10^5^ cells/mL were seeded in 6-well cell culture plates, where they were incubated for 24 h. After incubation, each plate was washed twice with 1500 µL of phosphate-buffered saline (PBS); a control group of cells was treated with DMEM basal medium (without FBS), while the experimental group was treated with DMEM basal medium supplemented with Forskolin (FSK) (MedChemExpress, Monmouth Junction, NJ, USA) and 3-Isobutyl-1-methylxanthine (IBMX) (Sigma-Aldrich, St. Louis, MO, USA), both drugs at a concentration of 0.5 mM. The two groups were treated for 96 h.

To confirm the neuroendocrine differentiation of A549 cells, they were analyzed under an inverted microscope. Photos of the cells were taken 0, 24, 48, 72, and 96 h after starting treatment; neurite-like processes were quantified with ImageJ’s Fiji software, using the NeuronJ plugin, tracing all the neurites present in 10 cells from each image. 

Identification of the neuroendocrine markers CgA and Syn was carried out using RT-PCR. For this, total RNA was extracted from A549 and A549_NED_ cells using the Direct-zol™ RNA Miniprep kit (Zymo Research, Irvine, CA, USA) following the manufacturer’s instructions. cDNA was synthesized using the RevertAid First Strand cDNA Synthesis Kit (Thermo Scientific, Waltham, MA, USA) combining random hexamers and oligo(dT)_18_ as primers. After determining nucleic acid concentration and quality, CgA, Syn, and GAPDH were amplified with PCR using specific primers for each gene (Table 2). PCR was performed according to the protocol described by the manufacturer of the main reagent, GoTaq Green Master Mix (Promega, Madison, WI, USA). The reaction consisted of an initial denaturation step of 5 min at 95 °C, followed by 30 repetitions of the following cycle: denaturation at 95 °C for 30 s, annealing at 55 °C for 30 s, and extension at 72 °C for 60 s. Reaction products were loaded onto a 2% agarose gel, which was run in an electrophoresis chamber for 45 min at 80 V. Finally, the gel was stained with ethidium bromide and developed on a UV transilluminator. Photographs of the gels were taken and analyzed with densitometry using Bio-Rad’s Quantity One Analysis Software V 4.6.8 (Hercules, CA, USA).

### 4.2. BALB/c Mice Immunization

Male 10–12-week-old BALB/c mice weighing 35 ± 5 g were used in this study. All handling, care, and use of animals for this research followed national and international guidelines regarding animal wellbeing, and the protocol was approved by the Chemistry Faculty Bioethics Committee from the Universidad Autónoma de Querétaro (CBQ21/125).

A total of 12 mice was divided into 3 groups (n = 4). The first group was a control, where mice received an injection of 100 µL of PBS (vehicle). The second group was immunized with A549 cells; in this group, mice received an injection of 5 × 10^5^ total cells in a final volume of 100 µL PBS. Finally, the third group was immunized with cells differentiated to a neuroendocrine phenotype (A549_NED_); mice in this group received an injection of 5 × 10^5^ cells in a final volume of 100 µL PBS. All injections were performed intraperitoneally. Cells were prepared for injection according to the protocol described in [79]. This immunization was performed 2 times, with one week between each injection. Seven days after the second immunization a blood sample was obtained through the tail vein for further analysis. Mice were sacrificed 21 days after the first immunization (14 days after the second immunization), recovering the blood and performing the extraction of mononuclear cells, keeping the plasma fraction for further analysis.

### 4.3. PBMC Extraction and Analysis with Flow Cytometry

Mononuclear cells were isolated using Histopaque 1083 (Sigma-Aldrich, St. Louis, MO, USA) according to the manufacturer’s instructions, using 1.5 mL of peripheral blood diluted 1:1 in PBS. Cells were resuspended in 500 µL of RPMI-1640 for maintenance or cytometry staining.

CD68 antibody anti-mouse coupled to PE-Vio615 (Miltenyi, Bergisch Gladbach, Germany; 130-112-674), CD4 antibody anti-mouse coupled to FITC (Miltenyi, 130-120-750), CD8a antibody anti-mouse coupled to PerCP-Vio700 (Miltenyi, 130-120-756), and CD335 antibody anti-mouse coupled to PE (Miltenyi, 130-112-201) were used for flow cytometry. The BD Cytofix/Cytoperm™ Fixation/Permeabilization Solution Kit (BD, Franklin Lakes, NJ, USA) was used to fix and permeabilize the cells. Washing steps and resuspension for analysis were performed in FACS buffer (PBS/EDTA/FBS). 

Flow cytometry was performed according to the manufacturer’s instructions, following Miltenyi’s cell surface flow cytometry staining protocol for CD4, CD8a, and CD335, as well as their intracellular flow cytometry staining protocol for CD68.

### 4.4. Serum Isolation and Analysis

To isolate serum, blood samples obtained after the second immunization were diluted 1:1 in PBS. These samples were centrifuged at 1000× *g* for 15 min, recovering only the upper fraction, which was stored at −70 °C until analysis.

ELISA kits for IL-2 (PeproTech, Cranbury, NJ, USA; 900-TM108), IL-10 (PeproTech 900-TM53), and IFN-γ (PeproTech 900-TM98) were used to determine cytokine levels in both serum and plasma, following the manufacturer’s instructions.

### 4.5. Co-Cultures between PBMCs and Cancer Cells 

A549 cells were seeded at a density of 1.5 × 10^5^ cells/mL in 12-well plates and neuroendocrine differentiation was induced as previously described. After treatment, undifferentiated and neuroendocrine cells were washed with PBS and PBMCs were seeded at a 1:2 ratio (A549:PBMCs). When pre-activated PBMCs were used, they were pre-incubated for 24 h with 1.5% PHA-M in RPMI 1640. All cocultures were maintained for 24 h in RPMI 1640 medium.

Cell viability was evaluated through a colorimetric assay and by direct cell counting with trypan blue for A549 cells and PBMCs, respectively. To do this, A549 cells were separated from the mononuclear cells by absorbing the medium (given that PBMCs are not adherent) and they were washed twice using PBS; then, a 1 mg/mL solution of MTT (Sigma-Aldrich) was added inside the wells. Plates were incubated for 2 h at 37 °C and then the formazan crystals were dissolved by adding isopropanol, shaking the plates for 30 min to allow their complete solubilization; finally, the absorbance was measured using spectrophotometry at 570 nm.

To determine PBMC viability, 100 µL aliquots from the cells in suspension that were removed by pipetting from the cocultures were diluted 1:2 in trypan blue solution (0.4%) and a direct cell count was performed using an automated cell counter (Corning, Corning, NY, USA).

### 4.6. Statistical Analysis 

All experiments were performed in triplicate, reporting the results as the mean ± SEM. In the experiments where two groups were evaluated, a comparison of means was performed using Student’s *t*-test, while in the experiments where three groups were compared, a one-way analysis of variance (ANOVA) was performed, followed by Tukey’s test for multiple comparisons of means. Differences with values of *p* < 0.05 were considered significant; however, in flow cytometric assays, due to the small population size (n = 4) and the complexity of the model, differences of *p* < 0.1 were also considered significant.

## 5. Conclusions

Mouse PBMCs induce A549 cell death, but they lose their cytotoxic activity when confronted with cancer cells that have acquired a neuroendocrine phenotype. Some immune cells die when confronted with A549 neuroendocrine cells, while in the presence of undifferentiated A549 cells, mononuclear cells proliferate and become activated. In vivo studies showed that there are significant differences between the profiles of circulating immune cells and cytokine profiles from mice immunized with A549 cells or with A549 neuroendocrine cells. Overall, this study suggests that neuroendocrine differentiation of lung adenocarcinoma cells induces an immunosuppressive response in mouse peripheral blood mononuclear cells.

## Figures and Tables

**Table 1:** Neurotransmitters and their effect on the immune system.

Neurotransmitter	Target Cell	Effect	Reference
Serotonin	Monocytes	Loss of ability to secrete proinflammatory cytokines such as TNF-α and IL-1β	[64]
	Macrophages	Suppresses IFN-γ-mediated phagocytosis as well as their ability to present antigens	[64]
	NK cells	Increased proliferative and migratory capacity	[64,65]
	T cells	Apoptosis, inhibition of PHA-mediated proliferation, reduced activation capacity, Th0 polarization towards Tregs	[66,67,68]
Dopamine *	Monocytes	Increased migration and adhesion	[69]
	Macrophages	Increased phagocytic activity	[70]
	NK cells	Increased cytotoxic activity against cancer cells	[71,72]
	T cells	Inhibition of apoptosis, increased cytokine expression (TNF-α, IL-10), increased migration and extravasation of CTLs	[70]
Norepinephrine	Monocytes	Impaired metabolism and cytokine production	[73]
	Macrophages	Impaired migration	[74]
	NK cells	Decreased activity	[75]
	T cells	Th2 polarization of immune response	[76]
	Cancer cells	Increased migratory potential	[77]

* Acting through D1 receptors.

**Table 2:** Primers used to identify neuroendocrine markers.

Gene	Sequence	Product (bp)
Syn	Fwd: 5′-AGACAGGGAACACATGCAAG-3′	123
	Rev: 5′-TCTCCTTAAACACGAACCACAG-3′	
CgA ^1^	Fwd: 5′-AACCGCAGACCAGAGGACCA-3′	102
	Rev: 5′-GTCTCAGCCCCGCCGTAGT-3′	
GAPDH	Fwd: 5′-TTGCCCTCAACGACCACTTT-3′	120
	Rev: 5′-TGGTCCAGGGGTCTTACTCC-3′	

^1^ Primer sequences obtained from [78].

## Data Availability

Not applicable.
